# Efficient Image-Only Inference for Multimodal Crop Disease Recognition via Modal Dropout and Adaptive Multi-Task Loss Learning

**DOI:** 10.3390/s26134052

**Published:** 2026-06-25

**Authors:** Jianlin Qiu, Depeng Gao, Shuxi Chen, Wenjie Liu

**Affiliations:** 1 School of Yonyou Digital Intelligence, Nantong Institute of Technology, Nantong 226002, China; qiujl@ntit.edu.cn (J.Q.); gaodepeng@ntit.edu.cn (D.G.); chenshq@ntit.edu.cn (S.C.); 2 School of Transportation and Civil Engineering, Nantong University, Nantong 226019, China

**Keywords:** crop leaf disease recognition, modal dropout, multi-task loss function, adaptive weight learning

## Abstract

Crop leaf diseases cause 10–40% annual yield losses, yet timely field diagnosis remains difficult. Vision-language models (VLMs) lift recognition accuracy with rich textual descriptions, but multimodal pipelines are too slow for real-time field use because they require text processing at inference. We present MTL-AWL, a framework built on a training–inference asymmetry: VLM text serves as privileged training-time supervision, and two coupled mechanisms—one retaining VLM semantics in the image encoder and one exploiting them—enable image-only deployment at multimodal accuracy. A modal-dropout strategy (p=0.6) intermittently masks the VLM text sequence during training, forcing the image encoder to retain cross-modal representations independently. An adaptive multi-task loss jointly optimizes InfoNCE contrastive alignment, attention diversity, and modality consistency under learnable softmax weights, consistently converging to a dominant contrastive weight (55% on soybean, 68% on PlantDoc)—identifying cross-modal alignment as the primary mechanism of VLM knowledge transfer. At inference, the model reaches 818 FPS (3.7× faster than multimodal methods) at only 0.41% accuracy cost, attaining 99.30%/98.89% (multimodal/image-only) on soybean and 72.65%/68.80% on PlantDoc—compact enough for real-time, offline field screening.

## 1. Introduction

Crops are major global sources of food, animal feed, and biofuel. Leaf diseases are among the most damaging threats to crop yields, causing annual losses of 10% to 40% in major production regions [1]. Climate change compounds the problem: warming temperatures and shifting rainfall patterns are altering pathogen distributions and increasing crop susceptibility across growing zones [2]. Identifying leaf diseases quickly and accurately is essential for limiting these losses and maintaining stable production.

Disease diagnosis today depends largely on visual inspection by trained experts, a process that is slow, labor-intensive, and inconsistent across different examiners. Scaling visual inspection across large farmlands is impractical. Deep learning, especially convolutional neural networks (CNNs), has improved automated disease recognition by learning visual features directly from images. Yet these models have practical limitations. They require large annotated datasets, which are scarce for less common or newly emerging diseases, and performance tends to drop when imaging conditions change or when models trained on one sensor are applied to another [3,4,5]. A more basic limitation is that single-modality methods use only visual information. When disease symptoms are ambiguous or subtle, images alone may not carry enough signal for reliable classification.

One response is to combine multiple data sources. Multimodal fusion methods pair images with textual symptom descriptions, often generated by large language models, to improve recognition performance [6,7]. The two modalities carry different information: images show the spatial appearance of lesions, while text describes symptom progression, distribution patterns, and environmental context that images alone do not capture. Combining them tends to improve classification accuracy, especially when training data is limited or comes from heterogeneous sources. But these gains depend on a loss function suited to multimodal learning. Most existing systems use standard cross-entropy loss, which does not explicitly align features across modalities, provides no regularization against overfitting, and cannot adjust the relative weight of different learning objectives on its own. A multi-task loss that jointly optimizes classification and cross-modal representation learning could close this gap.

Even so, multimodal methods are difficult to deploy for real-time use in the field. The bottleneck is inference speed: most multimodal models need text input at inference time, adding processing overhead. CMDF-VLM [8], for example, reaches only 222 FPS because of its text pipeline, while image-only models exceed 800 FPS. This 3.7× throughput gap matters when the target is real-time screening in resource-limited field settings. The loss function is the other bottleneck. Existing systems generally use a single cross-entropy loss that neither aligns cross-modal features nor balances multiple objectives automatically. In our experiments, replacing it with a weighted multi-task loss improved accuracy by 2.03%. The two bottlenecks are connected. Dropping the text modality at inference removes the cross-modal signal, making the loss function even more consequential: the model must learn discriminative features from images alone. A framework that handles inference efficiency and loss design together stands a better chance of working in practice.

We present MTL-AWL (Multi-Task Loss with Adaptive Weight Learning), a framework designed to fully exploit and retain the semantic knowledge provided by vision-language models. VLMs such as GLM-4.6 V generate rich textual descriptions from disease images—covering lesion morphology, color-texture patterns, and symptom progression—that substantially enhance recognition accuracy. However, two challenges remain: how to design a loss function that optimally extracts knowledge from VLM descriptions, and how to retain this knowledge at deployment without VLM inference. MTL-AWL addresses both challenges through a modality-aware training strategy (*p* = 0.6) that forces the image encoder to independently retain VLM-provided semantics, and an adaptive multi-task loss whose learned weights reveal that contrastive alignment is the primary mechanism for VLM knowledge transfer. At deployment, the model runs at 818 FPS on images alone.

MTL-AWL rests on a training-inference asymmetry: VLM-generated text serves as privileged training-time supervision, while two coupled mechanisms—one to retain VLM semantics in the image encoder, one to exploit them—enable image-only deployment that preserves multimodal accuracy. The main contributions of this paper are summarized as follows:

(1) VLM Knowledge Retention via Modality-Aware Training: We introduce a modality-aware training strategy that intermittently masks the convolved VLM-generated text sequence during training, so that cross-attention and fusion receive zero text input simultaneously, forcing the image encoder to independently retain VLM-enriched representations rather than relying on textual shortcuts.
(2) Adaptive Multi-Task Loss for VLM Knowledge Exploitation: We design a multi-task loss that jointly optimizes InfoNCE contrastive alignment, attention diversity, and modality consistency under softmax-normalized learnable weights. Convergence demonstrates that contrastive alignment predominates, confirming cross-modal alignment as the primary driver of VLM knowledge transfer.

The remainder of this paper is structured as follows. Section 2 provides a review of related works. Section 3 outlines our proposed methodology. In Section 4, we present and analyze the experimental results. Section 5 discusses the validity and robustness of our approach. Finally, Section 6 presents our conclusion.

## 2. Related Works

This section reviews the relevant literature on convolutional neural networks for crop disease recognition, multimodal data fusion for crop disease recognition, multi-task loss function design, and vision-language models.

### 2.1. Convolutional Neural Network for Crop Disease Recognition

CNNs are now the most widely used architecture for automated crop disease diagnosis. Geetharamani et al. [9] trained a CNN to detect foliar diseases from leaf images, while Barbedo [10] classified individual lesions rather than whole leaves. Liu et al. [11] adapted a stochastic residual network to estimate disease severity, and Chen et al. [12] designed LeafNet for tea plant pathology. Several other architectures have been tested as well: dual-network models for tomato diseases [13], DenseNet-121 variants tuned for apple leaf disorders [14], and side-by-side comparisons of deep learning against traditional machine learning [15].

Lightweight models are now common in disease recognition systems built for efficient field deployment. ShuffleNet variants [16,17], MobileNet [18,19], and GhostNet [20] give a reasonable baseline, but their feature extraction is coarse enough that various groups have modified them for agricultural use. A separate issue is the training objective: most CNN-based crop disease classifiers use a single cross-entropy loss, which handles neither class imbalance nor the combination of multiple learning goals.

### 2.2. Multimodal Data Fusion for Crop Disease Recognition

Several recent papers combine multiple data modalities for crop disease identification. Ametefe et al. [21] paired deep transfer learning with multimodal inputs, relying on data augmentation and improved feature extraction for leaf disease recognition. J. Zhou [22] built ITK-Net, an image-text system that integrates agricultural domain knowledge to produce more interpretable disease classifications. H. Li [23] proposed AMMFNet, which fuses RGB and multispectral images through attention mechanisms for apple disease and pest detection, reporting higher accuracy than single-source input. H. Lee [24] used visual and environmental data together for crop classification, disease identification, and severity estimation, with a multimodal augmentation component that raised accuracy by 2.58% over visual-only input. X. Liu [25] released the CDDM dataset for agricultural diagnostics along with a LoRA-based finetuning method for adapting multimodal models to farming tasks.

These methods share a practical drawback: they need text input during inference. The text preprocessing step adds enough latency to rule out real-time use in the field. Modality masking has been explored for general multimodal robustness [26]. Our approach applies a similar principle to a different goal: retaining VLM-generated semantic knowledge in the image encoder for efficient image-only deployment, rather than general modality robustness. The training paradigm also relates to the LUPI framework [27], where privileged information enriches training but is unavailable at inference; in our case, the privileged modality is automatically generated by a VLM rather than provided by a human teacher. Crucially, we pair masking with a loss built to maximize cross-modal knowledge transfer, so the retained VLM semantics drive image-only performance. This distinguishes MTL-AWL from classical teacher–student distillation [28], generalized distillation [29], and modality hallucination [30]: rather than distilling logits, we fix VLM semantics into the image encoder via contrastive alignment, so the novelty lies in coupling VLM-as-teacher training with an adaptive loss that concentrates on this alignment, not in dropout or weighting alone.

### 2.3. Multi-Task Loss Function Design

Standard cross-entropy loss works well on balanced datasets but is less effective when class frequencies are skewed. Lin et al. [31] proposed focal loss to handle this: by reducing the contribution of easy examples, the optimizer spends more time on hard cases. The method is now widely used in detection and classification tasks where some classes are much rarer than others.

Label smoothing [32] addresses a different problem. Instead of one-hot targets, it assigns a small probability to all classes, producing a softened distribution that keeps the model from becoming overconfident. The effect is better generalization, which matters in agricultural settings where disease appearance varies across cultivars, growth stages, and imaging conditions.

Contrastive learning has shifted how models learn representations from paired data. InfoNCE loss [33], widely used in self-supervised and multimodal learning, increases similarity between matched pairs while reducing it for mismatched pairs. In agricultural applications, this gives a way to align visual and textual features without relying on explicit class labels.

Multi-task learning (MTL) trains one model on several related tasks, sharing representations across them. The difficulty is assigning weights to each task’s loss. Kendall et al. [34] treated task weights as learnable parameters derived from per-task uncertainty, so no manual tuning is needed. GradNorm [35] adjusts weights dynamically to balance gradient magnitudes across tasks. Both approaches have been applied in general vision and language settings, but they have not been applied to maximize the exploitation of VLM-generated semantic features for agricultural disease recognition, where the loss function must simultaneously handle cross-modal knowledge extraction, feature diversity, and inter-modal consistency.

### 2.4. Vision-Language Model

Vision-language models that process both images and text have drawn increasing attention [36,37,38]. BLIP [39,40], DALL-E [41,42], GPT-4 [43], and ZhipuAI’s Longwriter [44] are among the widely used architectures. BLIP, for example, connects a pretrained vision encoder to a frozen large language model, transferring visual knowledge into the language model without retraining it.

In agricultural settings, these models have mostly been applied to caption generation and visual question answering on crop disease images. The outputs have encouraged further work on using these models to produce textual descriptions from visual inputs. Dense text prompts generated from disease images can help downstream tasks like classification, because they encode semantic information that pixel data alone does not provide.

Our framework builds on this direction. During training, modal dropout randomly drops the text modality so the model learns to function without it at inference time. An adaptive multi-task loss jointly optimizes classification accuracy, contrastive alignment, and regularization using learned per-task weights. The trained model receives vision-language supervision but requires only images at deployment, avoiding the text processing overhead that makes existing multimodal approaches too slow for real-time use.

## 3. Methodology

**Figure 1:**
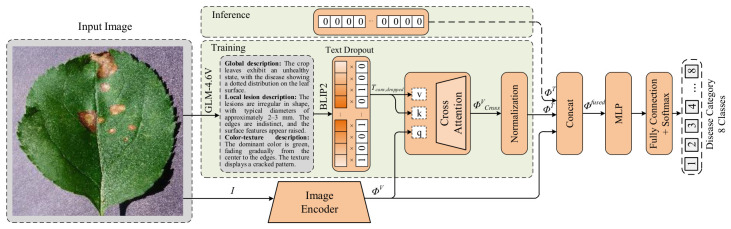
Workflow for our MTL-AWL framework. During training, both image and text features are extracted and fused through cross-attention for multi-modal learning. During inference, the text encoder is bypassed and text features are replaced with zero vectors, enabling efficient image-only classification while maintaining multi-modal learning benefits.

This section details the Multi-Task Loss Function Framework with Adaptive Weight Learning (MTL-AWL), which integrates visual features from crop disease images with semantic textual descriptions. The framework employs a training-inference asymmetry design: during training, it utilizes multi-modal inputs with modal dropout strategy and adaptive multi-task loss optimization; during inference, it performs efficient image-only classification. As shown in Figure 1, our method comprises two distinct workflows: Training Flow (with multi-modal input and comprehensive loss computation) and Inference Flow (with image-only input for efficient deployment).

### 3.1. Training Flow

The Training Flow represents the complete multi-modal learning pipeline where the model learns from both visual and textual modalities. This workflow enables the model to develop robust feature representations while maintaining the ability to perform image-only inference during deployment.

#### 3.1.1. Multi-Modal Feature Extraction

**Image Encoder.** The image encoder serves as the fundamental component for extracting discriminative visual features from soybean leaf disease images. In this work, we select ShuffleNetV2 as the backbone network for visual feature extraction, motivated by its superior trade-off between recognition accuracy and computational efficiency. The image encoder processes the input image $I\in R^{B\times 3\times H\times W}$, where *B* is the batch size and H×W represents the spatial dimensions, and produces visual features $\Phi_{raw}^{V}\in R^{B\times d_{img}}$, where $d_{img}$ is the ShuffleNetV2 output dimension. Subsequently, a projection layer maps the raw features to a unified hidden dimension:

(1) $$\Phi^{V}=\mathrm{LayerNorm}\left(\mathrm{GELU}\left(\mathrm{Linear}\left(\Phi_{raw}^{V},W_{proj}\right)\right)\right)$$

where $W_{proj}$ represents the learnable parameters of the projection layer, and $d_{h}$ denotes the hidden dimension for feature alignment across modalities.

**Text Encoder.** The textual description *T* is generated offline using GLM-4.6 V, a sophisticated vision-language model, which produces structured textual representations containing global description, local lesion description, and color-texture description. These descriptions are then processed by BLIP2’s pretrained text encoder to derive semantic features $T_{raw}\in R^{B\times L\times d_{BLIP2}}$, where *L* is the text sequence length and $d_{BLIP2}=768$ is the BLIP2 feature dimension. A text processing module with 1D convolution transforms the raw text features:

(2) $$T_{conv}=\mathrm{Conv}1D\left(T_{raw}\right)$$

where $T_{conv}\in R^{B\times L\times d_{h}}$ denotes the convolved text features, which will be passed through modal dropout before entering cross-attention and the aggregation layer.

#### 3.1.2. Modality-Aware Training for VLM Knowledge Retention

A key challenge in leveraging VLM-generated knowledge is ensuring that the image encoder can function independently at deployment. We address this by intermittently masking VLM-generated text features during training, so the model learns to predict from visual input alone. Crucially, dropout is applied to the convolved text sequence $T_{conv}$ rather than the aggregated vector, ensuring that both the cross-attention module and the fusion layer receive zero text input simultaneously.

Each training sample goes through one of two paths, controlled by the dropout probability $p_{dropout}$:

- Full Modal (probability $1-p_{dropout}$): Both visual features $\Phi^{V}$ and the full text sequence $T_{conv}$ are utilized for cross-attention and fusion, enabling the model to learn cross-modal correlations.
- Text Dropout (probability $p_{dropout}$): The text sequence is replaced with zero vectors 0, forcing both the cross-attention and fusion layers to rely solely on visual information.

Gradient flow. When text is dropped, the loss $L_{CE}=CE\left(fusion\left(\left[\Phi_{cross}^{V};0\right]\right),y\right)$ backpropagates through the image encoder only. Because $T_{conv}$ is zeroed before entering cross-attention, neither the attention weights nor the fusion layer receive any text signal—the model must map visual features to predictions on its own. The fusion layer learns to adjust:

(3) $$\Phi^{fused}\approx \left\{\begin{array}{cc} w_{1}\cdot \Phi_{cross}^{V}+w_{2}\cdot \Phi^{T} & \mathrm{if}\;\mathrm{text}\;\mathrm{available}\\ w_{img}\cdot \Phi^{V} & \mathrm{if}\;\mathrm{text}\;\mathrm{dropped} \end{array}\right.$$

where $w_{1}$, $w_{2}$, $w_{img}$ are learned via backpropagation and settle to different values depending on which modality is active.

Modal dropout. Applied to the convolved text sequence:

(4) $$T_{conv,dropped}=\left\{\begin{array}{cc} 0 & \mathrm{if}\;r<p_{dropout}\\ T_{conv} & \mathrm{otherwise} \end{array}\right.$$

where r∼U(0,1) is a random variable sampled from uniform distribution. The dropped sequence $T_{conv,dropped}$ is then passed to both the cross-attention module and the text aggregation layer. The aggregated text feature is computed as:

(5) $$\Phi^{T}=\mathrm{LayerNorm}\left(\mathrm{GELU}\left(\mathrm{Linear}\left(\mathrm{mean}\left(T_{conv,dropped},dim=1\right),W_{txt}\right)\right)\right)$$

When $T_{conv,dropped}=0$, the mean pooling naturally yields $\Phi^{T}=0$, so the fusion layer processes $\left[\Phi_{cross}^{V};0\right]$ without any separate masking. We implement modal dropout by randomly setting the text feature vector to zero with probability p during training. Through ablation studies (Section 4.2.4), we found *p* = 0.6 to be optimal, balancing multimodal performance and image-only robustness.

#### 3.1.3. Cross-Modal Feature Alignment

As shown in Figure 1, the cross-modal feature alignment module uses cross-attention to let text features condition the visual representation. The text acts as a guide, telling the model which visual patterns matter for crop disease recognition.

The module has $H_{att}$ heads. Visual features $\Phi^{V}\in R^{B\times d_{h}}$ are reshaped into $\Phi_{seq}^{V}\in R^{B\times 1\times d_{h}}$ as the Query(Q), and the dropout-adjusted text features $T_{conv,dropped}\in R^{B\times L\times d_{h}}$ supply both Key(K) and Value(V). Each text token weights a different part of the visual feature through this setup. The attention is computed as:

(6) $$\begin{array}{cc} \Phi_{cross}^{V} & =\mathrm{LayerNorm}(\Phi_{seq}^{V}+\mathrm{CrossAtt}\left(\Phi_{seq}^{V},T_{conv,dropped},T_{conv,dropped}\right))\\ \; & =\mathrm{LayerNorm}\left(\Phi_{seq}^{V}+\sum_{h=1}^{H_{att}}\mathrm{Softmax}\left(\frac{Q_{h}K_{h}^{⊤}}{\sqrt{d_{k}}}\right)V_{h}\right) \end{array}$$

where $Q_{h}=\Phi_{seq}^{V}W_{h}^{Q}$, $K_{h}=T_{conv,dropped}W_{h}^{K}$, $V_{h}=T_{conv,dropped}W_{h}^{V}$ for head *h*, and $d_{k}=d_{h}/H_{att}$. The output passes through a residual connection and layer normalization, giving $\Phi_{cross}^{V}\in R^{B\times d_{h}}$. Because the attention weights come from text features, the resulting visual features are biased toward regions that match disease descriptions. When modal dropout activates and $T_{conv,dropped}=0$, the cross-attention output reduces to the identity (residual only), so $\Phi_{cross}^{V}\approx \Phi^{V}$.

#### 3.1.4. Feature Fusion and Classification

The visual and textual features are concatenated along the feature dimension to form $\left[\Phi_{cross}^{V};\Phi^{T}\right]\in R^{B\times 2d_{h}}$, then projected through a linear layer:

(7) $$\Phi^{fused}=\mathrm{LayerNorm}\left(\mathrm{GELU}\left(\mathrm{Linear}\left(\left[\Phi^{V}cross;\Phi^{T}\right],Wfusion\right)\right)\right)$$

where [·;·] is concatenation and $W_{fusion}$ is the fusion layer parameters. A two-layer classifier maps the fused features to disease predictions:

(8) $$\hat{y}=\mathrm{Softmax}\left(Linearout\left(\mathrm{ReLU}\left(Linearhidden\left(\Phi^{fused}\right)\right)\right)\right)$$

where *C* is the number of disease categories. Keeping the fusion module to a single projection layer reduces parameter count, which matters for efficient deployment under limited memory and compute budgets.

### 3.2. Inference Flow

The Inference Flow represents the simplified deployment pipeline where the model performs efficient image-only classification. This workflow eliminates the dependency on text input during inference, making it suitable for real-time field applications.

#### 3.2.1. Image-Only Feature Extraction

During inference, only the image encoder is activated to process the input image. The text encoder is completely bypassed, eliminating the computational overhead associated with text processing. The visual features are extracted following the same procedure as in the training flow:

(9) $$\Phi^{V}=\mathrm{LayerNorm}\left(\mathrm{GELU}\left(\mathrm{Linear}\left(F_{encoder}\left(I,W_{conv}\right),W_{proj}\right)\right)\right)$$

#### 3.2.2. Bypass Text Processing

To achieve efficient image-only inference, the text processing pipeline is completely bypassed. Equivalently, this corresponds to setting the modal dropout probability to $p_{dropout}=1$ during training: the convolved text sequence is replaced with zero vectors, $T_{conv,dropped}=0$, which in turn yields $\Phi^{T}=0$ through the aggregation layer. Consequently, the cross-attention module produces only the residual (identity) output, effectively skipping cross-modal enhancement. This design significantly reduces computational overhead during inference, enabling real-time disease detection at low computational cost.

#### 3.2.3. Direct Classification

With text features bypassed, the fusion layer directly processes the visual features without cross-modal enhancement. The model effectively operates as a pure image classification pipeline during deployment:

(10) $$\Phi^{fused}=\mathrm{LayerNorm}\left(\mathrm{GELU}\left(\mathrm{Linear}\left(\left[\Phi^{V};0\right],W_{fusion}\right)\right)\right)$$

(11) $$\hat{y}=\mathrm{Softmax}\left({\mathrm{Linear}}_{out}\left(\mathrm{ReLU}\left({\mathrm{Linear}}_{hidden}\left(\Phi^{fused}\right)\right)\right)\right)$$

The training-inference asymmetry allows the model to leverage rich semantic information during training while achieving fast, image-only inference during deployment. Thanks to the modal dropout training strategy, the model develops robust visual feature representations that do not rely on text supervision, resulting in a minimal performance gap between multimodal training and image-only inference modes.

### 3.3. Adaptive Multi-Task Loss Computation

The adaptive multi-task loss computation framework integrates three complementary loss components that work synergistically to enhance model performance. The multi-task loss function integrates InfoNCE contrastive learning, attention diversity, and modality consistency to address various challenges in multi-modal disease recognition.

The total loss is formulated as:

(12) $$L_{total}=\sum_{i=1}^{3}w_{i}L_{i}$$

where $w_{i}$ represents the adaptive weight for loss component $L_{i}$.

#### 3.3.1. InfoNCE Contrastive Loss

We implement contrastive learning to achieve effective cross-modal feature alignment. This loss brings matching visual-textual pairs closer while pushing non-matching pairs apart:

(13) $$L_{InfoNCE}=-\frac{1}{N}\sum_{i=1}^{N}\log\frac{\exp(\mathrm{sim}\left(f_{i}^{V},f_{i}^{T}\right)/\tau )}{\sum_{j=1}^{N}\exp\left(\mathrm{sim}\left(f_{i}^{V},f_{j}^{T}\right)/\tau \right)}$$

where *N* is the batch size, sim(·,·) denotes cosine similarity, τ=0.07 is the temperature parameter, and $f_{i}^{V},f_{i}^{T}$ are the projected visual and textual features for sample *i*. The contrastive loss plays a crucial role in aligning visual and textual features in a shared embedding space, ensuring that $\Phi^{V}\approx \Phi^{T}$ for semantically corresponding image-text pairs. This alignment allows visual features to partially substitute for textual features when the latter are unavailable during inference, as the image encoder has learned to produce features that occupy similar regions in the embedding space as their textual counterparts.

#### 3.3.2. Attention Diversity Loss

We minimize the similarity between different attention heads to prevent redundancy and encourage diverse feature learning:

(14) $$L_{div}=\frac{2}{H(H-1)}\sum_{h=1}^{H}\sum_{h^{\prime }=h+1}^{H}\mathrm{cosine}\left(A_{h},A_{h^{\prime }}\right)$$

where *H* is the number of attention heads, and $A_{h}\in R^{h\times w}$ represents the attention map for head *h*.

#### 3.3.3. Modality Consistency Loss

We enforce consistency between cross-attention enhanced visual features and original textual features to maintain information integrity across modalities:

(15) $$L_{cons}={∥\Phi_{cross}^{V}-\Phi^{T}∥}_{2}^{2}$$

#### 3.3.4. Adaptive Weight Learning

The adaptive weight learning module dynamically adjusts the importance of different loss components during training. To maximize the utilization of VLM-provided semantic features, we parameterize the loss weights as trainable variables that adapt through backpropagation. The weights are normalized using softmax to ensure stable optimization:

(16) $$w=\mathrm{Softmax}\left(w_{learnable}\right)=\frac{\exp(w_{i})}{\sum_{j=1}^{3}\exp\left(w_{j}\right)}$$

where $w_{learnable}\in R^{3}$ are trainable parameters optimized through backpropagation.

Through our experiments, we observed that the adaptive learning mechanism automatically identifies contrastive learning as the most important loss component, assigning it the highest weight (55.0%), followed by modality consistency and attention diversity losses. This validates our hypothesis that cross-modal feature alignment is the most critical aspect of multimodal soybean leaf disease recognition.

### 3.4. Crop Disease Multimodal Dataset Construction

**Figure 2:**
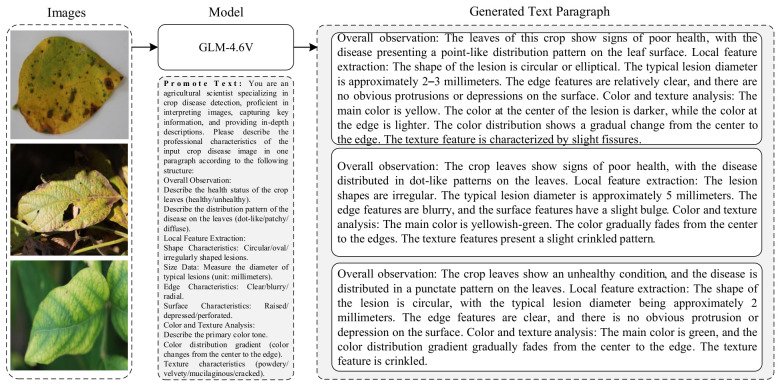
The crop disease multimodal dataset creation process.

We tested our framework on the Soybean Disease [45] and PlantDoc Dataset [46]. To build paired image-text data, we fed each disease image into GLM-4.6 V and collected the generated annotation. The prompts (Figure 2) were refined with plant pathologists to focus on the visual features that matter for diagnosis.

We set temperature and top-p both to 0.3 and capped output at 350–400 characters. All descriptions were generated through the official GLM-4.6 V API rather than a locally deployed checkpoint. Running the same image more than once gave nearly identical text, so we skipped post-processing entirely. Each prompt asks about the leaf’s overall condition, where lesions appear and what shape they take, and what color and texture the affected tissue has. We did not manually correct or remove any annotations—a deployed system would face the same raw outputs without human cleanup, so training on unfiltered text is the more realistic condition. Once generated, each description was encoded into a fixed-dimensional feature vector by a locally deployed BLIP-2 model, and the embeddings were cached locally to avoid repeated inference; during training, only these pre-extracted feature vectors are loaded, bypassing repeated GLM-4.6 V and BLIP-2 inference and substantially reducing computational overhead.

**Figure 3:**
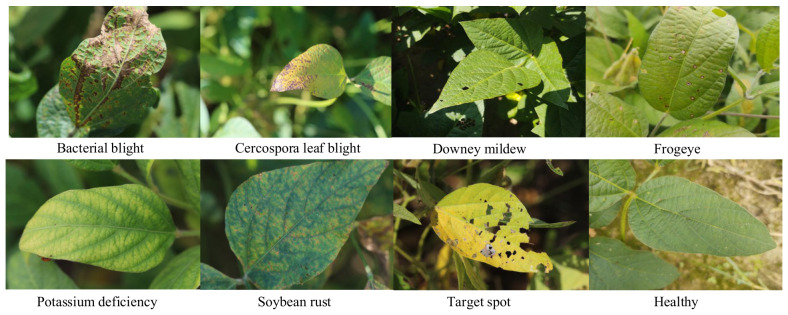
The example images from the soybean disease dataset.

The final soybean dataset has 10,722 image-text pairs across eight categories (healthy leaves plus diseases like bacterial blight and potassium deficiency), split 7:3 into 7505 training and 3217 evaluation samples. Figure 3 shows representative examples. In contrast, the PlantDoc dataset consists of 2552 image–text pairs covering 27 diverse categories of healthy and diseased plants across multiple crop species, partitioned at an approximately 9:1 ratio into 2316 training and 236 test samples, serving as a challenging benchmark for fine-grained disease identification under real-world conditions.

## 4. Experiment and Analysis

This section begins by detailing the framework and implementation of the multimodal agricultural datasets used in our experiments. We then conduct a series of ablation studies on these datasets to evaluate how the integration of textual features affects model performance, and benchmark the results against state-of-the-art image-only recognition methods.

### 4.1. Implementation Details

Our MTL-AWL uses a hidden dimension of 256 with 4 cross-attention heads and a modal dropout rate of 0.6, the latter chosen through ablation experiments. Training uses Adam (lr 0.001, weight decay 0.0001) with a batch size of 64 for 200 epochs, halving the learning rate every 50 epochs.

We apply standard data augmentation to the soybean images during training: random rectangular crops with aspect ratios between 3:4 and 4:3, covering 8% to 100% of the original area, resized to 224 × 224, followed by random horizontal flips and normalization. At test time, images are resized to 256 × 256 and a center 224 × 224 crop is used for classification.

All experiments were run on a single NVIDIA RTX 4090 (24 GB VRAM), with an AMD Ryzen 7 3700X CPU and 64 GB RAM. The framework is implemented in PyTorch 2.6.0.

### 4.2. Ablation Studies

We run ablation experiments along four dimensions: (1) the effect of different text descriptions, (2) progressive addition of loss functions, (3) fixed versus adaptive loss weights, (4) dropout probability, and (5) fusion architecture comparison.

#### 4.2.1. Impact of Text Descriptions on Model Performance

To isolate the contribution of each text channel, we add them one at a time on the soybean disease and PlantDoc datasets. The model takes three text inputs: a global description of the leaf’s overall condition, a description of lesion location and shape, and a description of color and texture in the affected tissue. The question is whether progressively richer text helps classification or whether a single description already suffices.

**Figure 4:**
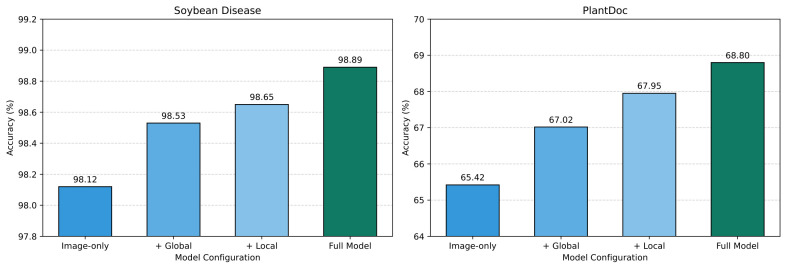
Impact of text descriptions on model performance for Soybean Disease and PlantDoc datasets.

Figure 4 shows the results. Starting from an image-only baseline of 98.12% accuracy, adding the global description brings it to 98.53%, then the lesion description to 98.65%. The full model with all three text inputs reaches 98.89%. Each text channel contributes a gain, though the increments shrink as more are stacked. The same trend holds on the PlantDoc dataset, with a larger absolute gain: accuracy rises from an image-only baseline of 65.42% to 67.02% with the global description, 67.95% with the lesion description, and 68.80% for the full model—an improvement of 3.38 percentage points versus 0.77 on soybean. The richer text supervision thus helps more on the harder PlantDoc benchmark, where the visual signal alone is less discriminative.

#### 4.2.2. Progressive Addition of Loss Functions

We progressively added loss components to a classification-only baseline—contrastive learning, then attention diversity, then modality consistency—to assess their individual and combined contributions.

As shown in Table 1, each component improves both image-only and multimodal accuracy on Soybean Disease, with contrastive learning giving the largest single gain (+1.10% image-only). The full combination reaches 98.89% image-only and 99.30% multimodal accuracy, narrowing the gap to 0.41%. On PlantDoc, the same scheme yields a smaller but consistent gain, the full model reaching 68.80% image-only and 72.65% multimodal accuracy. The multimodal–image-only gap, however, stays above 3.4% throughout—far larger than on soybean—indicating that the visual branch alone is less discriminative and the model relies more heavily on textual semantics on this harder benchmark.

#### 4.2.3. Fixed Versus Adaptive Weight Configuration

We compared several fixed weight configurations—equal [1.0, 1.0, 1.0], contrastive-enhanced [2.0, 0.5, 0.5], attention-enhanced [1.0, 1.5, 1.0], and consistency-enhanced [1.0, 0.5, 1.5]—against an adaptive weight learning mechanism.

As shown in Table 2, the adaptive mechanism outperforms every fixed configuration on Soybean Disease, reaching 98.89% image-only and 99.30% multimodal accuracy (gap 0.41%) versus 98.75%/99.30% (gap 0.55%) for the best fixed setup (consistency-enhanced). It learns weights of [0.55, 0.18, 0.27] for [contrastive, attention, consistency], assigning the largest share (55%) to contrastive learning. On PlantDoc, the adaptive scheme again leads on both modes (68.80% image-only, 72.65% multimodal), with weights converging to [0.68, 0.07, 0.23]—an even larger share (68%) for contrastive alignment and near-suppression of attention diversity (7%)—reinforcing that cross-modal alignment dominates on this harder benchmark. The gap stays around 3.8–4.0% across all configurations, far larger than on soybean.

**Figure 5:**
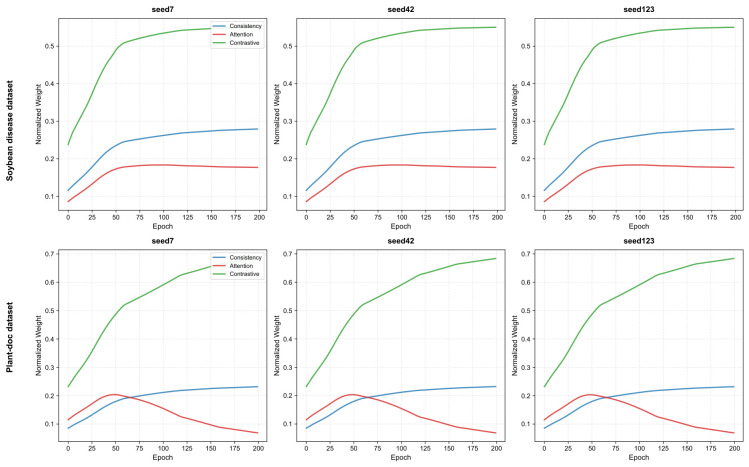
Convergence of the adaptive loss weights under different random seeds on the Soybean Disease and PlantDoc dataset.

The adaptive mechanism automatically identifies each component’s importance and adjusts it during training, consistently assigning the greatest weight to cross-modal alignment (contrastive learning)—validating our hypothesis on the centrality of image–text feature alignment. This weighting is also reproducible rather than seed-dependent: as Figure 5 shows, the learned weights converge to essentially the same distribution across different random seeds, matching the values reported in Table 2.

#### 4.2.4. Text Dropout Ablation Study

**Figure 6:**
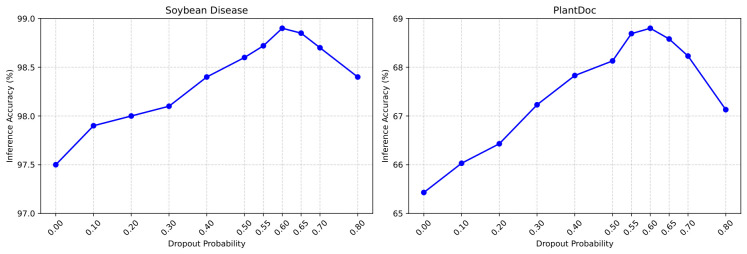
Text dropout ablation study: inference accuracy trend across different dropout probabilities on the Soybean Disease and PlantDoc datasets. The optimal performance is achieved at dropout probability p=0.6, reaching 98.89% accuracy on the Soybean Disease dataset and 68.80% on the PlantDoc dataset.

We varied the modal dropout probability from 0.0 to 0.8 to balance image-only inference robustness with multimodal benefits; Figure 6 shows the accuracy trend.

Without dropout (p=0.0), the model reaches only 97.52% image-only accuracy on the soybean disease dataset, indicating over-reliance on text. Accuracy rises with dropout, peaks at p=0.6 (98.89%, a 0.41% gap to multimodal inference), then degrades for p>0.6 (98.65% at p=0.7, 98.45% at p=0.8). PlantDoc shows the same trend over a wider range: accuracy rises from 65.42% (p=0.0) to a peak of 68.80% at p=0.6 (gap 3.85%), then falls to 68.23% (p=0.7) and 67.13% (p=0.8). The much larger residual gap reflects PlantDoc’s heavier reliance on textual semantics, yet the optimum remains p=0.6, showing the strategy transfers across datasets of differing difficulty. Thus p=0.6 gives the best balance—robust image-only inference while retaining multimodal supervision—whereas excessive dropout hurts. This calibrated dropout is what enables accurate real-time, text-free inference.

#### 4.2.5. Fusion Architecture Comparison

To verify that our fusion design is not an arbitrary choice, we compared it against two alternatives: the reversed attention direction and simple concatenation without cross-attention. All other components were held fixed—backbone, $d_{h}=256$ with four attention heads, p=0.6 modal dropout, and the adaptive multi-task loss—and image-only inference accuracy serves as the primary comparison. The experimental results are reported in Table 3.

Our design is optimal among the alternatives. The V → T direction outperforms the reversed T → V direction on image-only inference by 0.40 and 0.31 points on the two datasets—as expected, since the text modality is dropped at deployment, enriching the visual representation transfers to image-only inference whereas enriching text does not. Simple concatenation is consistently worse still, trailing cross-attention by roughly 0.4–0.7 points on both inference modes and both datasets.

### 4.3. Image Classification on the Soybean Disease and PlantDoc Datasets

To evaluate the efficacy and versatility of our approach, benchmark tests were conducted against leading models such as ResNet variants (18, 50) [47], MobileNet editions (v1 [18], v2 [19]), GhostNet [20], ShuffleNet-v2 [17], and CMDF-VLM [8].

The MTL-AWL framework delivers best performance on Soybean Disease and PlantDoc datasets (Table 4). GLM-4.6 V and the text encoder stay frozen, serving only to generate textual descriptors during training and adding no inference cost. The image-only configuration reaches 98.89% accuracy—higher than every image-only baseline and even the multimodal CMDF-VLM (98.74%), despite using no text at inference—while the full multimodal model reaches 99.30%. Notably, however, the CMDF-VLM model is highly parameter-efficient, with only 1.2 M parameters. The compact efficiency-oriented networks are also outperformed: MobileNet-v2, GhostNet, and the deeper ResNet-50 all trail our image-only model, which attains this at 6.07 M parameters—below the 11–24 M of the ResNet family—showing that VLM-distilled representations extract more discriminative power from a moderate backbone than the baselines obtain from either aggressive compression or larger capacity. The same advantage carries over to PlantDoc, a 27-class benchmark of field-condition leaf images that is substantially harder than the lab-style soybean set: image-only accuracy reaches 68.80% and the full multimodal model 72.65%, again ahead of every image-only baseline (strongest: MobileNet-v2, 67.95%) at 6.08 M parameters, confirming that the VLM-distilled representation generalizes to visually diverse real-field conditions rather than only the cleaner soybean images. This stems from three design choices: modal dropout (p=0.6) forces robust text-independent visual features; the adaptive multi-task loss balances the contrastive, attention-diversity, and consistency terms; and the ShuffleNet-v2 backbone deploys efficiently at 596.91 M FLOPs and 818 FPS (∼3.7× faster than CMDF-VLM).

**Figure 7:**
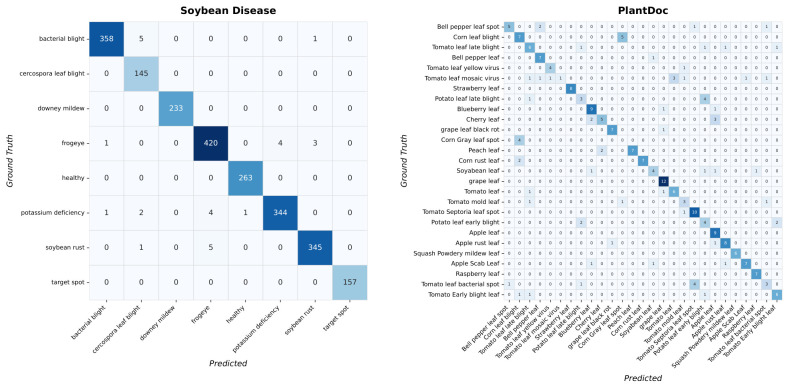
Confusion matrix of the Soybean Disease and PlantDoc datasets.

The confusion matrices (Figure 7) corroborate these results. On Soybean Disease, classification is near-perfect, with most categories above 99% recall and the only notable confusion between bacterial blight and cercospora leaf blight, which share early-stage symptoms. PlantDoc is markedly harder: its 27 field-condition classes leave a 3.85-point multimodal–image-only gap (68.80% vs. 72.65%), and residual errors concentrate among visually similar leaves (e.g., corn leaf blight vs. gray leaf spot) rather than signaling a representation failure; even so, MTL-AWL leads every PlantDoc baseline (strongest image-only baseline: MobileNet-v2, 67.95%) without a speed penalty, running at 826 FPS on par with ShuffleNet-v2 and ResNet-50.

## 5. Discussion and Analysis

The results in Table 4 show that MTL-AWL reaches high accuracy on the soybean disease and PlantDoc datasets. We trace this outcome to two design decisions. Modal dropout randomly withholds text during training so that the model learns to depend on visual features alone; the adaptive loss allocator, meanwhile, adjusts the relative weight assigned to contrastive learning, attention diversity, and modality consistency throughout optimization. The following subsections examine each mechanism in turn and discuss what the findings mean for real-time deployment in the field.

Effectiveness of Modal Dropout Training. Multimodal models often depend on text input at inference time. On Soybean Disease, the model trained without dropout (p=0.0) reached only 97.52% image-only accuracy, a 1.75% gap to the multimodal setting, showing that the visual branch was underutilized; modal dropout at p=0.6 closed this gap to 0.41% (98.89%). The same effect holds on PlantDoc, where dropout lifted image-only accuracy from 65.42% (p=0.0) to 68.80% (p=0.6), with the optimum again at p=0.6; its residual multimodal–image-only gap is larger (3.85%), reflecting that field-condition images are visually harder and the model leans more on textual semantics, yet dropout still transfers most of the multimodal gain to image-only inference. Randomly withholding text during training forces reliance on visual representations and regularizes the joint feature space, making image-only inference feasible without discarding multimodal gains.

VLM Knowledge Utilization and the Role of Contrastive Alignment. The adaptive weights reveal how VLM semantics are exploited: on Soybean Disease the contrastive loss alone takes 55% of the total weight, with attention diversity (18%) and modality consistency (27%) playing complementary roles—the former preventing redundant attention patterns and the latter keeping cross-attention-enhanced visual features aligned with the original VLM semantics. On PlantDoc, the contrastive share rises further to 68% while attention diversity is nearly suppressed (7%, consistency 23%), so both datasets consistently identify cross-modal alignment as the dominant channel of VLM knowledge transfer, and the harder benchmark leans on it even more heavily. The InfoNCE loss learns a shared embedding in which matched image–text pairs are pulled together, letting textual semantics disambiguate ambiguous visual cues; because modal dropout fixes these VLM-derived benefits into the image encoder, they persist at inference even without text, yielding a practical form of knowledge distillation from a large VLM to a lightweight model.

## 6. Conclusions

This study presented MTL-AWL, a framework that maximizes the exploitation and retention of vision-language model (VLM)-generated semantic knowledge for efficient crop leaf disease recognition. While our previous work (CMDF-VLM) demonstrated that VLM-generated text improves recognition accuracy, it required text input at deployment (222 FPS). MTL-AWL addresses this limitation through two key innovations: an adaptive multi-task loss that optimally extracts VLM knowledge through contrastive alignment (55% adaptive weight), attention diversity, and modality consistency; and a modality-aware training strategy that forces the image encoder to independently retain VLM-provided semantics, enabling image-only inference at 818 FPS—3.7× faster than CMDF-VLM. On the Soybean Disease dataset and the PlantDoc benchmark, MTL-AWL achieves 99.30% multimodal accuracy and 98.89% image-only accuracy on soybean, and 72.65% and 68.80% respectively on PlantDoc, with only 6.07 M parameters, demonstrating that VLM semantic knowledge can be effectively distilled into lightweight model for real-time field screening without VLM inference, text preprocessing, or internet connectivity. A useful direction going forward is few-shot adaptation to newly emerging pathologies; testing on other crops and across more diverse deployment conditions would also clarify how broadly the framework transfers.

## Figures and Tables

**Table 1:** Progressive addition of loss functions and their impact on model performance. The performance gap is calculated as the difference between multimodal and image-only inference accuracy.

Experiment	Dataset	Image-Only (%)	Multimodal (%)	Gap (%)
Baseline (Classification)	Soybean disease	96.90	98.00	1.10
+Contrastive		98.00	99.00	1.00
+Attention		98.60	99.25	0.65
+Consistency		98.89	99.30	0.41
Baseline (Classification)	PlantDoc	67.09	70.51	3.42
+Contrastive		68.38	71.79	3.41
+Attention		68.45	72.10	3.65
+Consistency		68.80	72.65	3.85

**Table 2:** Comparison of fixed and adaptive weight configurations for multi-task loss optimization on the Soybean Disease and PlantDoc datasets. Weights are represented as [contrastive, attention, consistency].

Configuration	Dataset	Weights	Image-Only (%)	Multimodal (%)	Gap (%)
Fixed (Equal)	Soybean disease	[1.0, 1.0, 1.0]	98.55	99.15	0.60
Fixed (Contrastive)		[2.0, 0.5, 0.5]	98.70	99.25	0.55
Fixed (Attention)		[1.0, 1.5, 1.0]	98.65	99.20	0.55
Fixed (Consistency)		[1.0, 0.5, 1.5]	98.75	99.30	0.55
Adaptive		[0.55, 0.18, 0.27]	98.89	99.30	0.41
Fixed (Equal)	PlantDoc	[1.0, 1.0, 1.0]	67.09	71.08	3.99
Fixed (Contrastive)		[2.0, 0.5, 0.5]	67.96	71.94	3.98
Fixed (Attention)		[1.0, 1.5, 1.0]	68.11	71.91	3.80
Fixed (Consistency)		[1.0, 0.5, 1.5]	68.35	72.17	3.82
Adaptive		[0.68, 0.07, 0.23]	68.80	72.65	3.85

**Table 3:** Comparison of fusion architectures on the Soybean Disease and PlantDoc datasets. “Cross-attn + Concat (V → T)” is our design (visual Query, textual Key/Value); “Cross-attn + Concat (T → V)” swaps Query and Key/Value; “Concat only” concatenates the two features without any cross-attention module. Image-only accuracy is the deployment metric. The “Concat only” variant, lacking an attention module, also forgoes the attention-diversity loss.

Variant	Dataset	Image-Only (%)	Multimodal (%)	Gap (%)
Cross-attn + Concat (V → T, Ours)	Soybean disease	98.89	99.30	0.41
Cross-attn + Concat (T → V)		98.55	99.06	0.51
Concat only (no cross-attn)		98.24	98.88	0.64
Cross-attn + Concat (V → T, Ours)	PlantDoc	68.80	72.65	3.85
Cross-attn + Concat (T → V)		68.49	72.14	3.65
Concat only (no cross-attn)		68.21	71.92	3.71

**Table 4:** Comparison of classification results with other state-of-the-art methods on the Soybean Disease and PlantDoc datasets. MTL-AWL results are reported as mean ± standard deviation over five independent repeated experiments.

Model	Dataset	Params ($\times 10^{6}$)	Acc (%)	Pre (%)	F1 (%)	R (%)	FLOPs	FPS
ResNet-18 [47]	Soybean disease	11.18	98.05	97.83	97.96	98.11	1.82 G	2066
ResNet-50 [47]		23.52	97.84	97.61	97.72	97.83	4.13 G	858
MobileNet-v2 [19]		2.23	97.71	97.60	97.64	97.69	326.24 M	979
GhostNet [20]		3.97	97.25	96.58	96.85	97.16	153.88 M	500
ShuffleNet-v2 [17]		4.72	97.95	97.78	97.83	97.89	151.71 M	840
CMDF-VLM (Multimodal) [8]		1.20	98.74	98.56	98.64	98.72	6.19 G	222
MTL-AWL (Multimodal)		6.60	99.30 ± 0.10	99.18 ± 0.12	99.33 ± 0.09	99.48 ± 0.08	598.94 M	718
MTL-AWL (Image-only)		6.07	98.89 ± 0.12	98.84 ± 0.13	98.95 ± 0.11	99.11 ± 0.10	596.91 M	818
ResNet-18 [47]	PlantDoc	11.19	66.67	68.46	64.44	65.59	1.82 G	2054
ResNet-50 [47]		23.56	67.09	68.32	65.35	66.50	4.13 G	847
MobileNet-v2 [19]		2.26	67.95	70.42	66.43	67.47	326.24 M	948
GhostNet [20]		3.99	65.81	64.74	63.22	65.29	153.88 M	494
ShuffleNet-v2 [17]		4.75	67.09	68.87	66.21	67.02	151.71 M	831
CMDF-VLM (Multimodal) [8]		1.23	68.76	70.59	66.84	67.59	6.19 G	214
MTL-AWL (Multimodal)		6.62	72.65 ± 0.12	73.90 ± 0.13	71.06 ± 0.11	71.31 ± 0.10	598.94 M	701
MTL-AWL (Image-only)		6.09	68.80 ± 0.14	70.63 ± 0.15	66.98 ± 0.13	67.77 ± 0.12	596.91 M	819

## Data Availability

The datasets utilized in this study are openly accessible. The soybean dataset is available at https://doi.org/10.5061/dryad.41ns1rnj3.
